# Antimicrobial-Resistant Infections in Hospitalized Patients

**DOI:** 10.1001/jamanetworkopen.2024.62059

**Published:** 2025-03-14

**Authors:** Hannah Wolford, Natalie L. McCarthy, James Baggs, Kelly M. Hatfield, Alexander Maillis, Babatunde Olubajo, Jonathan Bishop, Mia Ferretti, Michael R. Craig, Shelley S. Magill, L. Clifford McDonald, Dawn M. Sievert, Maroya Spalding Walters, John A. Jernigan, Joseph D. Lutgring, Sujan C. Reddy

**Affiliations:** 1 Division of Healthcare Quality Promotion, Centers for Disease Control and Prevention, Atlanta, Georgia

## Abstract

**Importance:**

Antimicrobial resistance is a major public health problem in the US. Estimating national rates of antimicrobial-resistant infections commonly associated with health care can aid in targeted public health efforts.

**Objective:**

To determine the national incidence rates of 6 pathogens over time: methicillin-resistant *Staphylococcus aureus* (MRSA), vancomycin-resistant *Enterococcus* spp (VRE), extended-spectrum cephalosporin-resistant *Escherichia coli* and *Klebsiella* spp (excluding *Klebsiella aerogenes)* (ESCR-EK), carbapenem-resistant *Enterobacterales* (CRE), carbapenem-resistant *Acinetobacter* spp (CRAsp), and multidrug-resistant (MDR) *Pseudomonas aeruginosa*.

**Design, Setting, and Participants:**

This cohort study used data from 2012 to 2022 on inpatient hospitalizations, clinical cultures, and facility-level characteristics. Hospital-months were included in the dynamic cohort if the hospital reported at least 1 culture with microbial growth accompanied by antimicrobial susceptibility testing (AST) results in the month. Data from the PINC-AI and Becton Dickinson Insights databases were used, and cases were defined as incident nonsurveillance cultures yielding an organism of interest with sufficient AST results for a phenotype of interest. Data were collected from January 2012 to December 2022 and analyzed from April 2023 to June 2024.

**Exposure:**

Inpatient hospitalizations with a discharge date in an included hospital month.

**Main Outcomes and Measures:**

National annual antimicrobial-resistant cases per 10 000 hospitalizations were obtained using weights based on facility-level characteristics. Cases were defined as community-onset if collected on or before day 3 of hospitalization and hospital-onset if obtained on day 4 or later.

**Results:**

This study cohort included 332 to 606 hospitals per year between 2012 to 2022 and 7 158 139 cultures. Antimicrobial-resistant pathogens accounted for an estimated 569 749 (95% CI, 475 949-663 548) cases and 179.6 (95% CI, 163.1-196.1) cases per 10 000 hospitalizations in 2022. Of these cases, 77% (437 657; 95% CI, 364 529-510 785) were community-onset and 23% (132 092; 95% CI, 108 241-155 943) were hospital-onset. MRSA (44% [251 854; 95% CI, 209 558-294 150]) and ESCR-EK (35% [200 884; 95% CI, 163 692-238 077]) made up the largest proportions of total infections in 2022, respectively. Rates of hospital-onset MRSA, VRE, CRE, CRAsp, and MDR *P aeruginosa* had periods of decline from 2012 to 2019; however, all pathogens experienced an increase in hospital-onset rates in 2020 and 2021. Community-onset ESCR-EK rates increased from 2012 to 2022, while community-onset rates of MRSA, VRE, and MDR *P aeruginosa* declined.

**Conclusions and Relevance:**

While antimicrobial resistance rates have experienced uneven declines in the US from 2012 to 2022, the burden of resistance remains substantial. These findings suggest that more effective strategies to reduce antimicrobial resistance are needed.

## Key Points

**Question:**

How has antimicrobial resistance changed from 2012 to 2022?

**Findings:**

In this cohort study of 7 158 139 cultures from 2012 to 2022, overall resistant cases per 10 000 hospitalizations declined between 2012 to 2016; however, progress varied across pathogens and was inconsistent before the COVID-19 pandemic. The pandemic was associated with notable increases in hospital-onset cases.

**Meaning:**

These findings suggest that innovative prevention interventions are needed to reduce antimicrobial resistance, as current interventions may be insufficient.

## Teaser

This cohort study examines incident rates of 6 antimicrobial-resistant pathogens among hospitalized patients in the US from 2012 to 2022.

## Introduction

Antimicrobial resistance is a major public health problem in the US and globally.^1,2,3,4^ The US Centers for Disease Control and Prevention (CDC) previously estimated that antimicrobial-resistant pathogens associated with health care (eg, methicillin-resistant *Staphylococcus aureus* [MRSA], vancomycin-resistant *Enterococcus* spp [VRE], extended-spectrum cephalosporin-resistant *Escherichia coli* and *Klebsiella* spp [excluding *Klebsiella aerogenes*] [ESCR-EK] suggestive of extended-spectrum β-lactamase production, carbapenem-resistant *Enterobacterales* [CRE], carbapenem-resistant *Acinetobacter* spp [CRAsp], and multidrug-resistant [MDR] *Pseudomonas aeruginosa*) caused more than 600 000 infections in patients who were hospitalized in the US in 2017.^1^ These antimicrobial-resistant pathogens are labeled as high priority by CDC and the World Health Organization.^4,5^ The COVID-19 pandemic disrupted many activities that could affect transmission of antimicrobial resistant pathogens. Understanding trends in antimicrobial resistance can help public health partners evaluate outcomes of current prevention interventions, such as contact precautions or antibiotic stewardship, and assess the need for additional preventive action. This report provides updated rate estimates for hospital-onset and community-onset cases of 6 antimicrobial-resistant pathogens in the US between 2012 and 2022.

## Methods

We followed the Strengthening the Reporting of Observational Studies in Epidemiology (STROBE) reporting guideline; institutional review board approval and informed consent were not required by the CDC as data were deidentified.^6^ We conducted a dynamic retrospective cohort study using inpatient hospitalization data from 2 data sources. PINC-AI (previously Premier) Healthcare Database (PHD) includes records for all discharges from participating acute care, general, and nonfederal US hospitals with a subset reporting microbiology data.^7^ The Becton Dickinson (BD) Insights Research Database is an electronic-based surveillance and clinical research database which provided select microbiology, patient and facility data.^8,9,10,11^ Methods are similar to our previous study.^1^ Hospital-months from January 2012 through December 2022 were included if the hospital reported at least 1 culture with microbial growth with accompanying antimicrobial susceptibility testing (AST) data during the month.

### Case Definition

We examined clinical cultures yielding an organism of interest that had accompanying AST results sufficient for determining an interpretation of susceptible, intermediate, or resistant for the phenotype of interest. Cultures without AST were excluded as a determination of resistant or susceptible could not be made. Organisms grown from clinical cultures collected from 3 days before admission to 3 days postdischarge were examined. We included only incident cultures, defined as cultures obtained from patients having no culture yielding organisms with the same phenotype in the previous 14 days.^12^ Patients with cultures from both normally sterile and nonsterile body sites yielding organisms with the same phenotype of interest obtained within 14 days of each other had only the sterile site culture result included. Cases were defined as incident clinical cultures that yielded an organism of interest with associated testing for a phenotype of interest (eTable 1 in Supplement 1). CRE and ESCR-EK organism definitions accounted for potential antimicrobial cascade reporting.^1^

Two infectious diseases physicians (J.D.L. and S.C.R.) performed a comprehensive review of the specimen body sites. Each specimen was categorized according to the anatomic site of collection (blood, respiratory, urine, other [eg, soft tissue or cerebrospinal fluid]), whether the specimen type was from a normally sterile (eg, blood or cerebrospinal fluid) or nonsterile body site, and whether the culture appeared to be obtained for surveillance purposes (eg, colonization screening cultures obtained from rectal, perirectal or nasal swabs). Surveillance cultures were excluded from the study. We defined community-onset cases as incident cultures collected before day 4 of hospitalization and hospital-onset cases obtained on day 4 or later.

### Statistical Analysis

To estimate the national annual rate of resistant cases, we used the American Hospital Association (AHA) database to obtain national inpatient hospitalizations stratified by facility characteristics (number of hospital beds, US census region, urban or rural designation, teaching status).^13^ We used iterative proportional fitting to generate hospital-specific weights to produce a distribution of hospitalizations that matched the national hospitalizations by facility characteristics.^14,15,16,17,18^ National estimates for antimicrobial-resistant cases and cases tested for resistance were calculated using a weighted means survey procedure. We calculated annual pooled rates using weighted estimates and hospitalizations. Percentage resistant was calculated as the weighted resistant cases divided by the weighted cases tested for resistance.

To obtain total resistant cases, we summed the resistant cases across all 6 pathogens. Because the same isolate could potentially count as a case for CRE and ESCR-EK phenotypes, we deduplicated cases prior to estimating the total national burden. Weighted national total resistant cases and 95% CIs were calculated using this deduplicated sum.

National rates were calculated annually for patient-level characteristics, specimen source, and organism species. Annual rates by facility characteristics were calculated using domain analyses to account for the survey methodology.^19^ Patient characteristics included age group and sex. Weighted rates for each facility or patient characteristic were calculated using the characteristic’s resistant cases and hospitalizations. Rates were censored if there were 10 or fewer unweighted resistant cases in the age group within the year.

We conducted a sensitivity analysis by restricting to facilities that reported data for 48 or more months (ie, 80% or more of all months) from January 2018 through December 2022 and extrapolated national resistant case rates using this consistent reporter cohort. These estimates were compared with our extrapolated, dynamic cohort estimates for 2018 to 2022.

Analyses were performed using SAS version 9.4 (SAS Institute) and R version 4.4.0 (R Project for Statistical Computing). We also used the PySpark version 3.5.0 (Apache Spark) programming interface in the statistical software Python 3.8.10 (Python Software Foundation) on the Microsoft Azure Databricks platform. This activity was reviewed by the CDC, deemed not research, and was conducted consistent with applicable federal law and CDC policy. Data were analyzed from April 2023 to June 2024.

## Results

Our study cohort included between 332 to 606 hospitals, representing 3 911 123 of 35 116 955 US hospitalizations (11%) to 6 365 375 of 34 427 965 US hospitalizations (18%) per year between 2012 to 2022 (Table 1 and Table 2). Most facilities were urban (eg, 334 of 495 hospitals [67%] to 256 of 332 hospitals [77%]), nonteaching (214 of 332 hospitals [64%] to 406 of 570 hospitals [71%]), and had less than 300 beds (202 of 332 hospitals [61%] to 404 of 570 hospitals [71%]) (Table 1 and Table 2). Facility-level characteristics differed between PHD, BD, and AHA; in 2022, 1790 of 4728 AHA hospitals (38%) were in the South vs 265 of 495 facilities in PHD and BD (54%) and 973 of 4728 AHA hospitals were in the West vs 36 of 495 facilities in PHD and BD (7%) (eTable 2 in Supplement 1). US hospitalizations decreased from 2020 to 2022, but the annual average number of patient-days per hospitalization in the study cohort increased from a low of 4.3 (26 589 677 patient days per 6 131 131 hospitalizations) in 2017 to 4.9 (19 109 930 patient days per 3 899 256 hospitalizations) in 2022 (Table 1 and Table 2). Of 8 995 403 clinical cultures with microbial growth for an organism of interest and AST results, 7 158 139 (83%) met the case definition (eFigure 1 in Supplement 1).

**Table 1.** Hospitals From the Study Cohort From 2012 to 2022

Characteristics	Hospitals, No. (%)										
	2012	2013	2014	2015	2016	2017	2018	2019	2020	2021	2022
Hospital-months	3542	4144	4196	4639	5267	6061	6393	6727	6415	5976	4970
No. of hospitals (% of total)											
Total No. of hospitals	332	370	405	440	499	575	601	606	592	570	495
Electronic database											
Becton Dickinison Insights Research Database	134 (40)	144 (39)	181 (45)	215 (49)	248 (50)	275 (48)	305 (51)	329 (54)	327 (55)	298 (52)	223 (45)
PINC-AI Healthcare Database	198 (60)	226 (61)	224 (55)	225 (51)	251 (50)	300 (52)	296 (49)	277 (46)	265 (45)	272 (48)	272 (55)
Location											
Urban	256 (77)	280 (76)	307 (76)	331 (75)	369 (74)	427 (74)	439 (73)	427 (70)	413 (70)	393 (69)	334 (67)
Rural	76 (23)	90 (24)	98 (24)	109 (25)	130 (26)	148 (26)	162 (27)	179 (30)	179 (30)	177 (31)	161 (33)
Teaching status											
Teaching	118 (36)	128 (35)	144 (36)	157 (36)	165 (33)	189 (33)	191 (32)	177 (29)	172 (29)	164 (29)	150 (30)
Nonteaching	214 (64)	242 (65)	261 (64)	283 (64)	334 (67)	386 (67)	410 (68)	429 (71)	420 (71)	406 (71)	345 (70)
No. of beds											
<300	202 (61)	228 (62)	252 (62)	283 (64)	329 (66)	379 (66)	403 (67)	420 (69)	415 (70)	404 (71)	350 (71)
≥300	130 (39)	142 (38)	153 (38)	157 (36)	170 (34)	196 (34)	198 (33)	186 (31)	177 (30)	166 (29)	145 (29)
US Census region											
Northeast	53 (16)	55 (15)	57 (14)	64 (15)	75 (15)	92 (16)	90 (15)	90 (15)	81 (14)	73 (13)	69 (14)
Midwest	67 (20)	85 (23)	96 (24)	116 (26)	129 (26)	141 (25)	143 (24)	145 (24)	133 (22)	134 (24)	125 (25)
South	169 (51)	184 (50)	199 (49)	213 (48)	237 (47)	279 (49)	304 (51)	309 (51)	315 (53)	301 (53)	265 (54)
West	43 (13)	46 (12)	53 (13)	47 (11)	58 (12)	63 (11)	64 (11)	62 (10)	63 (11)	62 (11)	36 (7)

**Table 2.** Hospitalizations From the Study Cohort From 2012 to 2022

Characteristics	Hospitalizations, No. (%)										
	2012	2013	2014	2015	2016	2017	2018	2019	2020	2021	2022
Age, y											
<1	421 195 (11)	472 016 (11)	478 474 (11)	506 707 (10)	558 928 (10)	607 693 (10)	622 659 (10)	604 325 (10)	543 789 (10)	490 552 (10)	406 957 (10)
1-17	159 185 (4)	162 725 (4)	163 659 (4)	166 926 (3)	165 356 (3)	167 178 (3)	165 503 (3)	158 960 (3)	108 474 (2)	102 084 (2)	93 063 (2)
18-54	1 387 540 (35)	1 497 410 (35)	1 522 804 (35)	1 665 699 (34)	1 868 066 (34)	2 029 637 (33)	2 072 590 (33)	2 016 170 (32)	1 767 388 (33)	1 612 930 (33)	1 232 330 (32)
55-64	556 451 (14)	617 431 (14)	639 763 (15)	725 125 (15)	840 304 (15)	939 754 (15)	980 343 (15)	971 660 (15)	827 198 (15)	744 810 (15)	559 162 (14)
65-74	569 260 (15)	646 178 (15)	660 263 (15)	757 437 (16)	897 191 (16)	1 035 217 (17)	1 091 948 (17)	1 111 123 (18)	943 669 (18)	870 350 (18)	686 877 (18)
≥75	808 362 (21)	886 556 (21)	875 711 (20)	999 546 (21)	1 156 796 (21)	1 346 382 (22)	1 429 255 (22)	1 436 100 (23)	1 172 082 (23)	1 095 325 (22)	920 408 (24)
Unknown	9130 (0.2)	7338 (0.2)	6467 (0.1)	5753 (0.1)	5896 (0.1)	5270 (0.1)	3077 (0)	3850 (0.1)	1951 (0)	815 (0)	459 (0)
Sex											
Male	1 639 541 (42)	1 809 563 (42)	1 839 464 (42)	2 059 393 (43)	2 358 137 (43)	2 648 273 (43)	2 765 384 (43)	2 755 120 (44)	2 387 941 (45)	2 185 250 (44)	1 718 920 (44)
Female	2 255 990 (58)	2 467 892 (58)	2 494 462 (57)	2 754 072 (57)	3 118 245 (57)	3 467 805 (57)	3 588 425 (56)	3 531 326 (56)	2 962 028 (55)	2 714 925 (55)	2 171 710 (56)
Annual cohort No. of hospitalizations^a^	3 911 123 (11)	4 289 654 (12)	4 347 141 (13)	4 827 193 (14)	5 492 537 (16)	6 131 131 (18)	6 365 375 (18)	6 302 188 (18)	5 364 551 (17)	4 916 866 (15)	3 899 256 (12)
Annual cohort No. of patient-days^a^	17 476 491 (9)	19 028 518 (10)	19 277 884 (11)	21 080 693 (11)	23 873 202 (13)	26 589 677 (14)	27 800 772 (15)	27 418 920 (15)	24 831 906 (14)	24 040 352 (13)	19 109 930 (10)
Cohort patient days per hospitalization^b^	4.5	4.4	4.4	4.4	4.3	4.3	4.4	4.4	4.6	4.9	4.9
Annual No. of US hospitalizations^c^	35 116 955	34 400 422	33 836 221	34 108 406	34 307 695	34 554 279	34 427 965	34 281 005	31 476 346	32 028 284	31 727 850
Annual No. of US patient-days^c^	187 208 529	184 347 424	182 713 687	184 466 419	185 426 378	185 636 360	184 713 538	184 748 009	174 947 622	184 611 675	186 676 126

^a^ Percentages were calculated using the annual American Heart Association (AHA) hospitalizations or patient-days as the denominator.

^b^ Cohort patient days per hospitalization are used to approximate the average annual inpatient length of stay and were calculated by dividing cohort patient days by cohort hospitalizations.

^c^ Data on all US hospitals (short-term, acute care) are from the AHA.

### National Estimates of Antimicrobial-Resistant Pathogens

**Figure 1.**
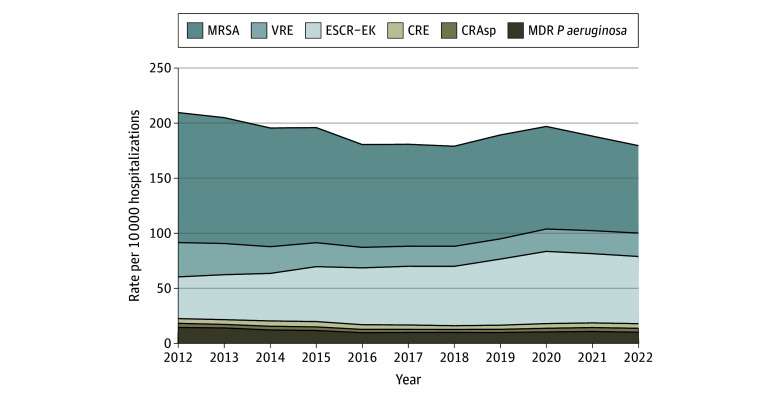
National Estimates of Antimicrobial-Resistant Cases Per 10 000 Hospitalizations Stratified by Year From 2012-2022 Total cases include methicillin-resistant *Staphylococcus aureus* (MRSA), vancomycin-resistant *Enterococcus* spp (VRE), extended-spectrum cephalosporin-resistant *Escherichia coli* and *Klebsiella* spp (excluding *Klebsiella aerogenes*) (ESCR-EK), carbapenem-resistant Enterobacterales (CRE), carbapenem-resistant *Acinetobacter* spp (CRAsp), and multidrug-resistant (MDR) *Pseudomonas aeruginosa.* As the same isolate could potentially count as a case for CRE and ESCR-EK phenotypes, we deduplicated cases for the rates of ESCR-EK that also met the CRE definition prior to estimating the total national rate. Total resistant case rates will not align with the sum of the 6 individual case rates. Estimates produced using inpatient hospitalization data from the PINC-AI Healthcare Database and the Becton Dickinson Insights Research Database.

Six pathogens accounted for an estimated 569 749 (95% CI, 475 949-663 548) resistant cases in 2022; more than 75% were community-onset resistant cases (437 657 [95% CI, 364 529-510 785] cases) and less than 25% were hospital-onset resistant cases (132 092 [95% CI, 108 241-155 943] cases) (eTable 3 and eFigure 2 in Supplement 1). The overall resistant cases per 10 000 hospitalizations decreased from 2012 (209.6 [95% CI, 197.0-222.1] cases per 10 000 hospitalizations) to 2016 (180.5 [95% CI, 167.7-193.4] cases per 10 000 hospitalizations) (Figure 1; eFigure 2 and eTable 4 in Supplement 1). Subsequently, the overall rate of resistant cases plateaued until 2018 (179.0 [95% CI, 168.9-189.2] cases), increased starting in 2019 (189.2 [95% CI, 178.8-199.7] cases), peaked in 2020 (197.0 [95% CI, 185.4-208.5] cases), and then declined in 2022 (179.6 [95% CI, 163.1-196.1] cases) with pathogen-specific trends varying over time (Figure 1).

### Hospital-Onset Pathogen Results

**Figure 2.**
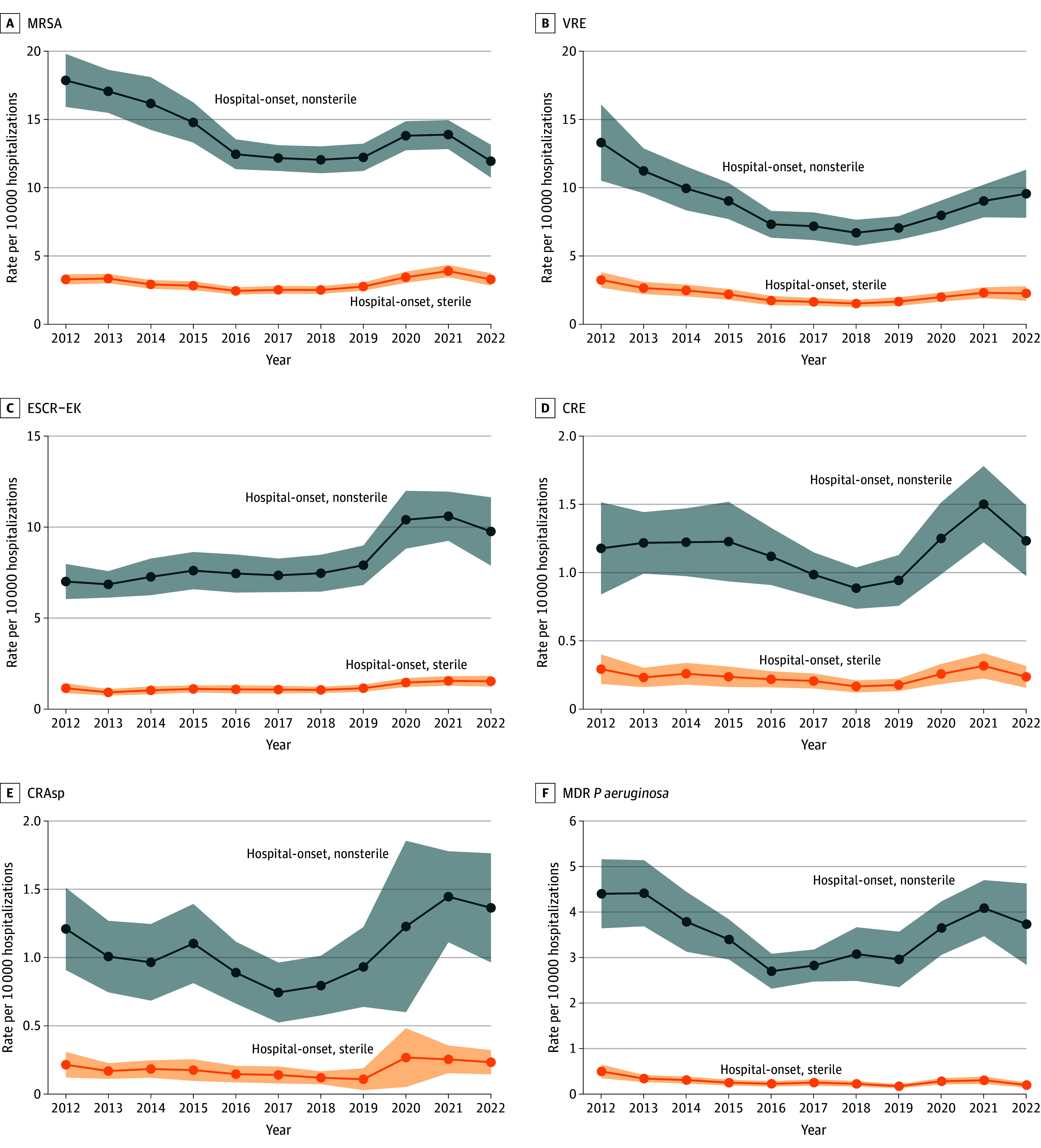
National Hospital-Onset Antimicrobial-Resistant Cases Per 10 000 Hospitalizations From 2012 to 2022, by Specimen Source Specimen source was stratified into nonsterile body sites (eg, urine, wound) and normally sterile body sites (eg, blood, cerebrospinal fluid). Estimates produced using inpatient hospitalization data from the PINC-AI Healthcare Database and the Becton Dickinson Insights Research Database. Shading indicates 95% CI. CRAsp indicates carbapenem-resistant *Acinetobacter* species; CRE, carbapenem-resistant *Enterobacterales*; ESCR-EK, extended-spectrum cephalosporin-resistant *Escherichia coli* and *Klebsiella* spp (excluding *Klebsiella aerogenes*); MDR, multidrug-resistant; MRSA, methicillin-resistant *Staphylococcus aureus* (MRSA); VRE, vancomycin-resistant *Enterococci*.

Hospital-onset resistant case rates declined from 2012 (48.6 [95% CI, 43.7-53.6]) to 2016 (34.7 [95% CI, 31.7-37.8]), followed by elevated levels in 2020 (42.7 [95% CI, 38.6-46.8]) to 2022 (41.6 [95% CI, 36.2-47.1]) (eFigure 2 and eTable 4 in Supplement 1). Every antimicrobial-resistant pathogen had increased hospital-onset sterile and nonsterile rates in 2020 and 2021 compared with 2018 and 2019; for example, hospital-onset MRSA sterile rates increased from a low of 2.5 (95% CI, 2.2-2.8) in 2018 to a high of 3.9 (95% CI, 3.5-4.3) in 2021 and hospital-onset ESCR-EK nonsterile rates increased from a low of 7.5 (95% CI, 6.5-8.5) in 2018 to a high of 10.6 (95% CI, 9.2-11.9) in 2021 (Figure 2 and eTable 5 in Supplement 2). Rates of resistant blood, urine, and respiratory cases varied by pathogen (eFigure 3 in Supplement 1 and eTable 6 in Supplement 2).

The percentage resistant for MRSA, VRE, CRE, CRAsp, and MDR *P aeruginosa* was stable or decreased from 2012 to 2022. Among hospital-onset *E coli* and *Klebsiella* spp, the percentage of ESCR-EK cases in nonsterile body sites increased from 12.2% (95% CI, 10.7%-13.6%) in 2012 to 19.7% (95% CI, 17.7%-21.7%) in 2022; and increased from 17.5% (95% CI, 14.4%-20.7%) to 24.5% (95% CI, 21.5%-27.4%) among normally sterile body sites (eFigure 4 in Supplement 1 and eTable 5 in Supplement 2).

There were differences in resistant cases per 10 000 hospitalizations by region and species. Among hospital-onset CRE, hospital-onset CR *K pneumoniae* rates decreased from 2012 to 2022 while hospital-onset CR *E cloacae* complex and *E coli* rates did not decrease (eFigure 5 in Supplement 1 and eTable 7 in Supplement 2). Hospital-onset CRE rates decreased in the Northeast US census region with stable or increased rates in other regions (eTable 8 in Supplement 2 and eFigure 6 in Supplement 1). Rates of hospital-onset MRSA, CRE, CRAsp, and MDR *P aeruginosa* were higher among males and all hospital-onset resistant case rates were higher in the subpopulations aged 55 to 64 years and 65 to 74 years compared with younger patients (eTable 9 in Supplement 2).

### Community-Onset Pathogen Results

**Figure 3.**
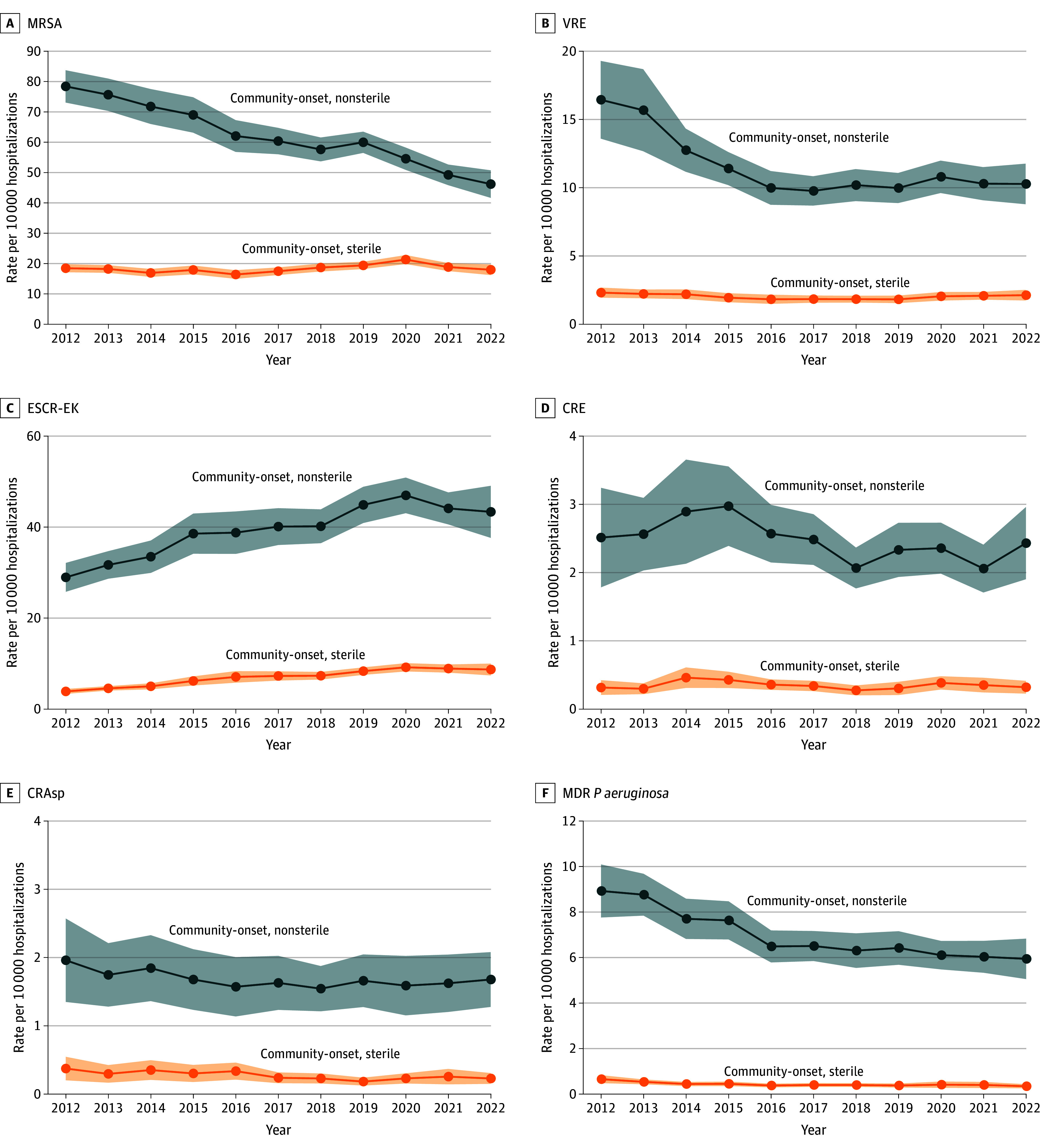
National Community-Onset Antimicrobial-Resistant Cases Per 10 000 Hospitalizations (2012-2022), by Specimen Source Specimen source was stratified into nonsterile body sites (eg, urine, wound) and normally sterile body sites (eg, blood, cerebrospinal fluid). Estimates produced using inpatient hospitalization data from the PINC-AI Healthcare Database (PHD) and the Becton Dickinson (BD) Insights Research Database. Shading indicates 95% CI. CRAsp indicates carbapenem-resistant *Acinetobacter* species; CRE, carbapenem-resistant *Enterobacterales*; ESCR-EK, extended-spectrum cephalosporin-resistant *Escherichia coli* and *Klebsiella* spp (excluding *Klebsiella aerogenes*); MDR, multidrug-resistant; MRSA, methicillin-resistant *Staphylococcus aureus* (MRSA); VRE, vancomycin-resistant *Enterococci*.

Community-onset resistant cases per 10 000 hospitalizations declined from 2012-2022 driven by declines in community-onset nonsterile MRSA, VRE, and MDR *P aeruginosa* (Figure 3; eFigure 2 and eTable 4 in Supplement 1). Community-onset ESCR-EK rates increased from 2012 through 2020 in normally sterile sites (3.9 [95% CI, 3.3-4.4] to 9.2 [95% CI, 8.3-10.1]) and nonsterile sites (28.9 [95% CI, 25.8-32.1] to 47.0 [95% CI, 43.1-50.9]) (eTable 5 in Supplement 2). Community-onset MRSA rates from nonsterile sites decreased from 78.4 (95% CI, 73.1 to 83.7) in 2012 to 46.2 (95% CI, 41.6 to 50.7) in 2022 with similar declines in respiratory and urine specimens; community-onset MRSA rates from normally sterile sites, largely blood specimens, were stable during this time (Figure 3; eFigure 7 in Supplement 1 and eTable 5 and eTable 6 in Supplement 2).

The percentage of ESCR-EK cases among community-onset *E coli* and *Klebsiella* spp from nonsterile body sites increased from 8.8% (95% CI, 8.0%-9.6%) in 2012 to 14.3% (95% CI, 13.0%-15.6%) in 2022. The percentage of ESCR-EK among community-onset *E coli* and *Klebsiella* spp sterile body sites increased from 7.1% (95% CI, 6.3%-8.0%) in 2012 to 13.7% in 2022 (95% CI, 12.2%-15.3%) (eFigure 8 in Supplement 1 and eTable 5 in Supplement 2).

Additional stratifications of resistant rates highlighted important trends in subpopulations. Community-onset ESCR *E coli* rates increased while community-onset ESCR *K pneumoniae* rates remained stable; community-onset CR *K pneumoniae* rates decreased while community-onset CR *E cloacae* complex and *E coli* rates did not (eFigure 5 in Supplement 1 and eTable 7 in Supplement 2). Community-onset VRE and CRE experienced early sharp declines in the Northeast region (2012 to 2015 for VRE; 2012 to 2019 for CRE), although both plateaued for the remainder of the study (eTable 8 in Supplement 2 and eFigure 9 in Supplement 1). Comparing patient characteristics, children aged 1 to 17 years had the largest absolute declines in community-onset MRSA rates from 2012 to 2022 (eTable 9 in Supplement 2).

### Sensitivity Analysis

The sensitivity analysis results from hospitals consistently reporting data from 2018 to 2022 did not differ substantially from the overall analysis findings. On average, the consistent reporter cohort had slightly higher estimates of resistant cases per 10 000 hospitalizations but lower precision (eFigures 10 and 11 in Supplement 1).

## Discussion

The burden of antimicrobial resistance in the US remains substantial, with approximately 570 000 resistant cases among hospitalized patients in 2022. There have been important changes in rates over the past decade as resistant cases per 10 000 hospitalizations for 5 of the 6 pathogens experienced declines, including declines in both hospital-onset and community-onset resistant cases. However, progress in reducing resistant case rates stalled or, for some pathogens, reversed even before the COVID-19 pandemic. This detailed description provides insights that can be used by health care partners to evaluate factors contributing to these trends and inform the development of novel approaches.

In 2020, we observed increases in hospital-onset resistant case rates like what others have found.^20,21,22,23^ Changes to health care use during the COVID-19 pandemic, such as decreases in overall admissions, surges in high-risk patients with COVID-19, and higher proportions of patients who were sicker and did not have COVID-19 contributed to increased hospital-onset resistant case rates.^20,24,25,26,27^ We found an increase in average patient days per hospitalization from 2020 to 2022. This suggests an increase in patients with severity of illness that continued when COVID-19 hospitalizations declined and may reflect larger trends in an aging US population with increased chronic illness or a move to manage less acute patients in outpatient care.^28^ Additionally, infection control practices like contact precautions for patients with some resistant pathogens and monitoring excess antimicrobial use decreased during the pandemic, which may have led to increased antimicrobial resistance.^29,30,31^ Our findings suggest a need for prevention interventions that can maintain effectiveness despite strain on health care systems and workers.

Overall community-onset rates declined during our study period. These declines were mostly driven by decreases in community-onset MRSA rates in nonsterile body sites, which aligns with previous studies.^1,32,33,34,35^ Community-onset MRSA rates from blood and normally sterile sites were stable or increased during this time, which suggests that declines in nonsterile MRSA may not be due to decreases in colonization prevalence. Changes in hospitalization patterns for patients with community-onset MRSA from nonsterile sites, such as improved emergency department or outpatient management, may be contributing to declines as patients with these infections may be less likely to be admitted.^36,37^ ESCR-EK was the only community-onset pathogen to increase from 2012 to 2022; these results are consistent with other studies.^38,39,40,41,42,43^ The ST131 *E coli* strain has been shown to be the principal strain responsible for this increase.^44,45,46,47^ The success of this strain may be due to antibiotic use or strain virulence characteristics.^45,48,49^ Additionally, community transmission may be increasing due to increasing ESCR *E coli* intestinal carriage among healthy individuals.^50^ ESCR-EK transmission may also be increasing in other health care settings, such as long-term care.^51^

We provided detailed stratified rates of resistant cases to inform health care partners of the pathogens and subpopulations most impacted by antimicrobial resistance and allow for targeted prevention activities. For instance, although CRE has been stable from 2012 to 2022, analyses by species show substantial declines in CR *K pneumoniae* but not in CR *E cloacae* complex or *E coli*. This pattern may be explained by reductions in ST258 carbapenemase-producing *K pneumoniae*.^52,53,54,55,56^ Community-onset MRSA rates stratified by age highlight dramatic decreases in rates among children. Previous work has shown decreases in pediatric MRSA and hypothesized that early recognition of noninvasive MRSA and increased clinician confidence has led to increased management in outpatient settings. Reductions in MRSA may also be due to better community infection control or changes in circulating MRSA strains.^57^

### Limitations

Our analyses have several limitations, some of which were previously reported.^1^ Data limitations did not allow for the determination of clinically diagnosed infections. Using cultures from nonsurveillance specimens vs patient’s diagnosis codes better capture resistant infections^58,59^; however, a subset of nonsterile cultures may not represent true infections. These cultures provide information on the epidemiologic burden of colonized patients, who serve as transmission reservoirs and carriers at risk of infection. Thus, they have been included in this and previous burden estimates.^1^ Additionally, trends in antimicrobial resistance may be impacted by variable culturing practices at hospitals, which we are unable to identify in our cohort. However, examinations of blood and cerebrospinal fluid culture test use in PHD indicated infection incidence was not driven by changes in testing.^60^

Classification of community-onset and hospital-onset was based on timing of specimen collection and does not imply acquisition in those settings. Community-onset cases could not be further classified into those with or without previous health care exposures due to limited information on prior inpatient and long-term care exposures. Culture-independent diagnostic tests (CIDT) (ie, tests without AST results) were not used in the generation of these estimates. For blood specimens, the US Food and Drug Administration recommends that CIDT be used in conjunction with traditional culture and AST.^61^ During our study, CIDT for additional specimen types have been approved and increasing use of CIDT may impact future studies.^62,63^ Additionally, cases were determined based on AST results at the clinical laboratory; therefore, changes in breakpoints over the study period may affect the number of resistant cases identified. Resistance detected at the clinical laboratory may not be confirmed at reference laboratories.^47,52^ We report clinical laboratory AST as clinicians use it to make treatment decisions. Finally, this analysis is limited to infections in the inpatient setting and does not include outpatient and long-term care settings.

Our study was based on a large but not randomly selected cohort of hospitals, allowing for the inclusion of a diverse population. Comparisons with other estimates provided strong evidence that these data are nationally representative.^32,44,64,65^ The dynamic cohort of our hospitals may be impacted by noncontinuous reporters (ie, hospitals who contribute only a subset of months of data to the analysis). We performed a subanalysis of hospitals consistently reporting from 2018 to 2022 to confirm a dynamic cohort did not introduce bias and did not find substantial changes from our main analysis. The strengths of our methods include weighted estimates that allow for discussion of burden and trends at a national level, the ability to stratify by patient and facility characteristics, and a longitudinal analysis that includes rates prior to, during, and postpandemic.

This analysis used 2 data sources, PHD and BD, vs the 3 data sources our group previously used to estimate national resistant case rates.^1^ This decreased the number of hospitals contributing to the analysis and required changes to our weighting by extrapolating to the US census region instead of census division. Reliance on electronic health databases for national estimation of antimicrobial resistance burden in the US may be unreliable as proprietary data products change or become unavailable over time. Our study only captures 11% to 18% of US hospitalizations, highlighting the need for more comprehensive antimicrobial resistance data. CDC’s National Healthcare Safety Network released the Antimicrobial Use and Resistance Module to provide a mechanism for facilities to conduct surveillance for antimicrobial use and pathogen resistance data.^66^ This module will allow facilities, networks, and public health officials to track antimicrobial resistance, benchmark against other facilities, and provide consistent data access over time.

## Conclusions

Antimicrobial resistance represents a threat to modern medicine^67^ and reducing antimicrobial resistance should remain a public health priority. We found uneven declines in rates throughout 2012 to 2022. While gains have been made in reducing community-onset rates, these declines were largely due to community-onset MRSA and were counterbalanced by increases in community-onset ESCR-EK. Increases in hospital-onset rates for all 6 pathogens in 2020 emphasized that prevention efforts may not be sufficient in times of additional health care strain. These analyses can inform health care partners of the pathogens and populations that can be prioritized for intervention to achieve the biggest impact in reducing antimicrobial resistance. Our findings underscore the need for innovative prevention strategies to reduce the burden of antimicrobial resistance as current interventions may be insufficient.

## Notes

Supplement 1. **eTable 1.** Detailed pathogen phenotype definitions **eTable 2.** Demographics for all included hospitals, stratified by data source (Becton Dickinson Insights Research Database and PINC-AI Healthcare Database, compared with the distribution of US hospitals as provided by the American Hospital Association, 2022 **eFigure 1.** Accounting of sample size among all positive cultures **eTable 3.** National estimates of 2022 antimicrobial-resistant cases: overall and by pathogen **eFigure 2.** National estimates of the sum of methicillin-resistant *Staphylococcus aureus*, vancomycin-resistant *Enterococcus *spp, extended-spectrum cephalosporin-resistant *Escherichia coli* and *Klebsiella *spp (excluding *Klebsiella aerogenes*) (ESCR-EK), carbapenem-resistant *Enterobacterales*, carbapenem-resistant *Acinetobacter *spp, and multidrug-resistant *Pseudomonas aeruginosa* stratified by location-onset and year with 95% CIs, 2012-2022 **eTable 4.** National sum of methicillin-resistant *Staphylococcus aureus*, vancomycin-resistant *Enterococcus *spp, extended-spectrum cephalosporin-resistant *Escherichia coli* and *Klebsiella *spp (excluding *Klebsiella aerogenes*) (ESCR-EK) suggestive of extended-spectrum β-lactamase production, carbapenem-resistant *Enterobacterales* (CRE), carbapenem-resistant *Acinetobacter *spp, and multidrug-resistant *Pseudomonas aeruginosa* cases per 10 000 hospitalizations **eFigure 3.** National estimated rates of hospital-onset (HO) antimicrobial-resistant cases per 10 000 hospitalizations, stratified by specimen body site (blood, urine, respiratory) and year with 95% CIs, 2012-2022 **eFigure 4.** The percent of hospital-onset (HO) resistant cases out of all HO cases tested for resistance, stratified by specimen body site (sterile or nonsterile) and year **eFigure 5.** National estimated rates of antimicrobial-resistant cases per 10 000 hospitalizations for carbapenem-resistant *Enterobacterales* (CRE), extended-spectrum cephalosporin-resistant *Escherichia coli* and *Klebsiella *spp (excluding *Klebsiella aerogenes*) (ESCR-EK) suggestive of extended-spectrum β-lactamase production, and vancomycin-resistant *Enterococcus* (VRE), stratified by species, location onset and year with 95% CIs, 2012-2022 **eFigure 6.** National estimated rates of hospital-onset (HO) antimicrobial-resistant cases per 10 000 hospitalizations by US census region, 2012-2022 **eFigure 7.** National estimated rates of community-onset (CO) antimicrobial-resistant cases per 10 000 hospitalizations, stratified by specimen body site (blood, urine, respiratory) and year, 2012-2022 **eFigure 8.** The percentage of community-onset (CO) resistant cases out of all CO cases tested for resistance, stratified by specimen body site (sterile or nonsterile) and year **eFigure 9.** National estimated rates of community-onset (CO) antimicrobial-resistant cases per 10 000 hospitalizations, by US census region, 2012-2022 **eFigure 10.** National estimated hospital-onset (HO) antimicrobial-resistant cases per 10 000 hospitalizations **eFigure 11.** National estimated community-onset (CO) antimicrobial-resistant cases per 10 000 hospitalizations

Supplement 2. **eTable 5.** National estimated rates of cases tested for resistance and antimicrobial-resistant cases per 10 000 hospitalizations with 95% CIs and percentage resistance, stratified by specimen body site (sterile or nonsterile), location onset and year, 2012-2022. **eTable 6.** National estimated rates of antimicrobial-resistant cases per 10 000 hospitalizations with 95% CIs and percentage of total pathogen rate stratified by specimen body sites (blood, urine, and respiratory), pathogen, location onset and year, 2012-2022 **eTable 7.** National estimated rates of antimicrobial-resistant cases per 10 000 hospitalizations for carbapenem-resistant *Enterobacterales* (CRE), extended-spectrum cephalosporin-resistant *Escherichia coli* and *Klebsiella* spp (excluding *Klebsiella aerogenes*) (ESCR-EK) suggestive of extended-spectrum β-lactamase production, and vancomycin-resistant *Enterococcus* (VRE), stratified by species, location onset and year, 2012-2022 **eTable 8.** National estimated rates of antimicrobial-resistant cases per 10 000 hospitalizations, stratified by facility characteristics, location onset and year, 2012-2022 **eTable 9.** National estimated rates of antimicrobial-resistant cases per 10 000 hospitalizations, stratified by patient characteristics, location onset and year, 2012-2022

Supplement 3.
Data Sharing Statement
