# Supplementary material for: Antimicrobial-Resistant Infections in Hospitalized Patients
Source: JAMA Netw Open. 2025 Mar 14;8(3):e2462059. doi: 10.1001/jamanetworkopen.2024.62059 (PMC11909612; doi:10.1001/jamanetworkopen.2024.62059)
Supplement: Supplement 1. — eTable 1. Detailed pathogen phenotype definitions eTable 2. Demographics for all included hospitals, stratified by data source (Becton Dickinson Insights Research Database and PINC-AI Healthcare Database, compared with the distribution of US hospitals as provided by the American Hospital Association, 2022 eFigure 1. Accounting of sample size among all positive cultures eTable 3. National estimates of 2022 antimicrobial-resistant cases: overall and by pathogen eFigure 2. National estimates of the sum of methicillin-resistant Staphylococcus aureus, vancomycin-resistant Enterococcus spp, extended-spectrum cephalosporin-resistant Escherichia coli and Klebsiella spp (excluding Klebsiella aerogenes) (ESCR-EK), carbapenem-resistant Enterobacterales, carbapenem-resistant Acinetobacter spp, and multidrug-resistant Pseudomonas aeruginosa stratified by location-onset and year with 95% CIs, 2012-2022 eTable 4. National sum of methicillin-resistant Staphylococcus aureus, vancomycin-resistant Enterococcus spp, extended-spectrum cephalosporin-resistant Escherichia coli and Klebsiella spp (excluding Klebsiella aerogenes) (ESCR-EK) suggestive of extended-spectrum β-lactamase production, carbapenem-resistant Enterobacterales (CRE), carbapenem-resistant Acinetobacter spp, and multidrug-resistant Pseudomonas aeruginosa cases per 10 000 hospitalizations eFigure 3. National estimated rates of hospital-onset (HO) antimicrobial-resistant cases per 10 000 hospitalizations, stratified by specimen body site (blood, urine, respiratory) and year with 95% CIs, 2012-2022 eFigure 4. The percent of hospital-onset (HO) resistant cases out of all HO cases tested for resistance, stratified by specimen body site (sterile or nonsterile) and year eFigure 5. National estimated rates of antimicrobial-resistant cases per 10 000 hospitalizations for carbapenem-resistant Enterobacterales (CRE), extended-spectrum cephalosporin-resistant Escherichia coli and Klebsiella spp (excluding Klebsiella aerogenes) (ESCR [file jamanetwopen-e2462059-s001.pdf]

Supplemental Online Content

Wolford H, McCarthy NL, Baggs J, et al. Antimicrobial-resistant infections in US hospitalized patients. *JAMA Netw Open*. 2025;8(2):e2462059. doi:10.1001/jamanetworkopen.2024.62059

eTable 1. Detailed pathogen phenotype definitions

eTable 2. Demographics for all included hospitals, stratified by data source (Becton Dickinson Insights Research Database and PINC-AI Healthcare Database, compared with the distribution of US hospitals as provided by the American Hospital Association, 2022

eFigure 1. Accounting of sample size among all positive cultures

eTable 3. National estimates of 2022 antimicrobial-resistant cases: overall and by pathogen

eFigure 2. National estimates of the sum of methicillin-resistant *Staphylococcus aureus*, vancomycin-resistant *Enterococcus* spp, extended-spectrum cephalosporin-resistant *Escherichia coli* and *Klebsiella* spp (excluding *Klebsiella aerogenes*) (ESCR-EK), carbapenem-resistant *Enterobacterales*, carbapenem-resistant *Acinetobacter* spp, and multidrug-resistant *Pseudomonas aeruginosa* stratified by location-onset and year with 95% CIs, 2012-2022

eTable 4. National sum of methicillin-resistant *Staphylococcus aureus*, vancomycin-resistant *Enterococcus* spp, extended-spectrum cephalosporin-resistant *Escherichia coli* and *Klebsiella* spp (excluding *Klebsiella aerogenes*) (ESCR-EK) suggestive of extended-spectrum β-lactamase production, carbapenem-resistant *Enterobacterales* (CRE), carbapenem-resistant *Acinetobacter* spp, and multidrug-resistant *Pseudomonas aeruginosa* cases per 10 000 hospitalizations

eFigure 3. National estimated rates of hospital-onset (HO) antimicrobial-resistant cases per 10 000 hospitalizations, stratified by specimen body site (blood, urine, respiratory) and year with 95% CIs, 2012-2022

eFigure 4. The percent of hospital-onset (HO) resistant cases out of all HO cases tested for resistance, stratified by specimen body site (sterile or nonsterile) and year

eFigure 5. National estimated rates of antimicrobial-resistant cases per 10 000 hospitalizations for carbapenem-resistant *Enterobacterales* (CRE), extended-spectrum cephalosporin-resistant *Escherichia coli* and *Klebsiella* spp (excluding *Klebsiella*

© 2025 Wolford H et al. *JAMA Network Open*.

aerogenes) (ESCR-EK) suggestive of extended-spectrum β-lactamase production, and vancomycin-resistant *Enterococcus* (VRE), stratified by species, location onset and year with 95% CIs, 2012-2022

eFigure 6. National estimated rates of hospital-onset (HO) antimicrobial-resistant cases per 10 000 hospitalizations by US census region, 2012-2022

eFigure 7. National estimated rates of community-onset (CO) antimicrobial-resistant cases per 10 000 hospitalizations, stratified by specimen body site (blood, urine, respiratory) and year, 2012-2022

eFigure 8. The percentage of community-onset (CO) resistant cases out of all CO cases tested for resistance, stratified by specimen body site (sterile or nonsterile) and year

eFigure 9. National estimated rates of community-onset (CO) antimicrobial-resistant cases per 10 000 hospitalizations, by US census region, 2012-2022

eFigure 10. National estimated hospital-onset (HO) antimicrobial-resistant cases per 10 000 hospitalizations

eFigure 11. National estimated community-onset (CO) antimicrobial-resistant cases per 10 000 hospitalizations

This supplemental material has been provided by the authors to give readers additional information about their work.

© 2025 Wolford H et al. *JAMA Network Open*.

eTable 1. Detailed pathogen phenotype definitions

Pathogen	Organisms Included in Definition	Antibiotics Included in Definition	Definition of Resistance Phenotype[a]	Denominator for Calculating Proportion of Isolates with Resistant Phenotype[a]
Methicillin-resistant *Staphylococcus aureus* (MRSA)	*Staphylococcus aureus*	methicillin, oxacillin, cefoxitin	Any isolate that tested (R) to at least 1 of these: methicillin, oxacillin, cefoxitin	Any isolate with at least 1 susceptible or non-susceptible result (S, I, R) to: methicillin, oxacillin, cefoxitin
Vancomycin-resistant *Enterococcus* (VRE)	*Enterococcus* spp.	Vancomycin	Any isolate that tested (R) to vancomycin	Any isolate that tested (S, I, R) to vancomycin
Carbapenem-resistant Enterobacterales (CRE)	*E. coli, Klebsiella* spp., *Enterobacter* spp.	imipenem, meropenem, doripenem, ertapenem, ampicillin, ampicillin/sulbactam, amoxicillin/clavulanic acid, piperacillin/tazobactam, cefazolin, cefoxitin, cefotetan	Any isolate with at least 1 resistant result (R) to imipenem, meropenem, doripenem, ertapenem	[b]Any isolate with at least 1 non-susceptible or susceptible result (S, I, R) to imipenem, meropenem, doripenem, ertapenem OR same isolate with at least 2 reported susceptible (S) results to: ampicillin, ampicillin/sulbactam, amoxicillin/clavulanic acid, piperacillin/tazobactam, cefazolin, cefoxitin, cefotetan
Extended-spectrum cephalosporin-resistant *E. coli* and *Klebsiella* spp. (ESCR-EK)	*E. coli, Klebsiella* spp. *(not Klebsiella aerogenes)*	cefotaxime, ceftriaxone, ceftazidime, cefepime, ampicillin, piperacillin, aztreonam, cefazolin	Any isolate with at least 1 non-susceptible, result (I or R) to: cefotaxime, ceftriaxone, ceftazidime, cefepime	[c]Any isolate with at least 1 susceptible or non-susceptible result (S, I, R) to: cefotaxime, ceftriaxone, ceftazidime, cefepime OR same isolate with at least 2 reported susceptible (S) results to: ampicillin, piperacillin, aztreonam, or cefazolin
Carbapenem-resistant *Acinetobacter* (CRAsp)	*Acinetobacter* spp.	imipenem, meropenem, doripenem	Any isolate with at least 1 non-susceptible result (I or R) to: imipenem, meropenem, doripenem	Any isolate with at least 1 susceptible or non-susceptible result (S, I, R) to imipenem, meropenem, or doripenem
Multidrug-resistant (MDR) *Pseudomonas aeruginosa*	*Pseudomonas aeruginosa*	1. Extended-spectrum cephalosporins (cefepime, ceftazidime), 2. Fluoroquinolones (ciprofloaxacin, levofloxacin), 3. Aminoglycosides (amikacin, gentamicin, tobramycin), 4. Carbapenems (imipenem, meropenem, doripenem), 5. Piperacillin Group (piperacillin, piperacillin/tazobactam)	Any isolate that tested either (I) or (R) to at least 1 drug in at least 3 of the medication categories	Any isolate with at least 1 susceptible or non-susceptible result (S, I, R) to at least 1 drug in the medication categories

© 2025 Wolford H et al. *JAMA Network Open*.

[a]Antimicrobial susceptibility testing result interpretations were categorized as susceptible (S), intermediate (I), resistant (R) or unknown. Unknown interpretations were excluded from the results. Less than 0.01% of interpretations were listed as susceptible dose-dependent or non-susceptible. Susceptible dose-dependent interpretations were recategorized as S; non-susceptible interpretations were recategorized as I.

[b]We accounted for cascade reporting by assuming Enterobacterales isolates to be carbapenem-susceptible if no carbapenem susceptibility result was reported but the isolate was reported to be susceptible to >1 of the following: ampicillin, ampicillin/sulbactam, amoxicillin/clavulanic acid, piperacillin/tazobactam, cefazolin, cefoxitin, or cefotetan.

[c]We accounted for cascade reporting by assuming Enterobacterales isolates to be susceptible to third and fourth generation cephalosporins if no susceptibility test results to these agents were reported but the isolate was reported to be susceptible to >1 of the following: ampicillin, piperacillin, aztreonam, or cefazolin.

© 2025 Wolford H et al. *JAMA Network Open*.

eTable 2. Demographics for all included hospitals, stratified by data source (Becton Dickinson (BD) Insights Research Database & PINC-AI Healthcare Database (PHD), compared with the distribution of U.S. hospitals as provided by the American Hospital Association (AHA), 2022

	All US hospitals (AHA)		PHD & BD combined		PHD		BD	
	Hospitals	%	Hospitals	%	Hospitals	%	Hospitals	%
Total	4,728		495		272		223	
Urban	2,898	61%	334	67%	176	65%	158	71%
Rural	1,830	39%	161	33%	96	35%	65	29%
Teaching	2,007	42%	150	30%	77	28%	73	33%
Non-teaching	2,721	58%	345	70%	195	72%	150	67%
No. of beds <300	3,910	83%	350	71%	199	73%	151	68%
No. of beds >=300	818	17%	145	29%	73	27%	72	32%
U.S. Census Region								
Northeast	568	12%	69	14%	27	10%	42	19%
Midwest	1,397	30%	125	25%	84	31%	41	18%
South	1,790	38%	265	54%	150	55%	115	52%
West	973	21%	36	7%	11	4%	25	11%
Annual Hospitalizations	31,727,850		3,899,256	12%	2,053,628	6%	1,845,628	6%

© 2025 Wolford H et al. *JAMA Network Open*.

eFigure 1. Accounting of sample size among all positive cultures

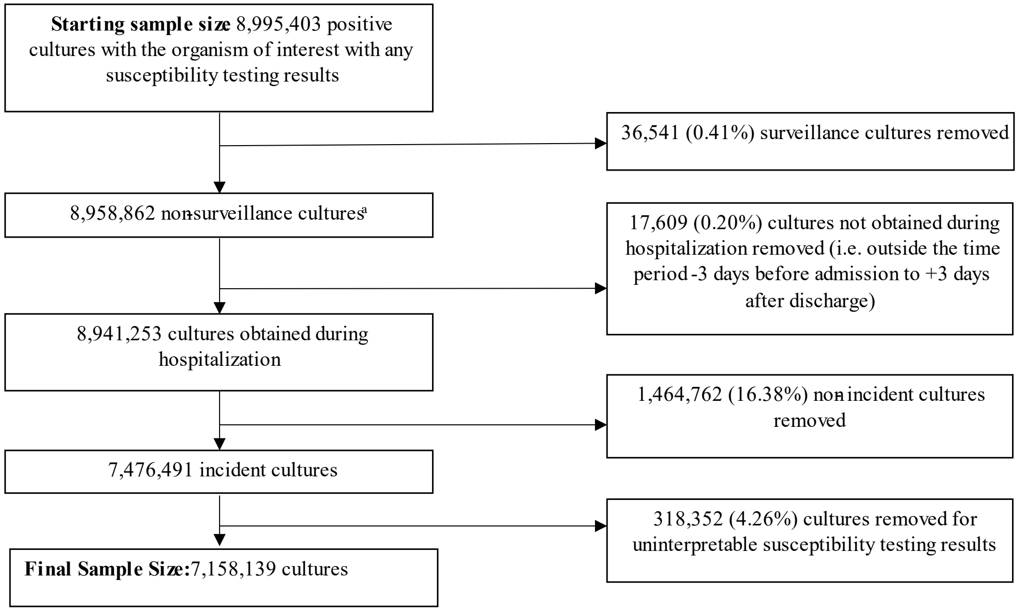

[a]Surveillance cultures were defined as cultures obtained from body sites typically used for colonization screening, such as rectal, perirectal or nasal swabs.

© 2025 Wolford H et al. *JAMA Network Open*.

eTable 3. National estimates of 2022 antimicrobial-resistant cases: overall and by pathogen, with 95% confidence intervals (CI)

	Overall		Hospital-onset		Community-onset	
	Estimate	95% CI	Estimate	95% CI	Estimate	95% CI
Total[a]	569,749	475,949 - 663,548	132,092	108,241 – 155,943	437,657	364,529 – 510,785
MRSA	251,854	209,558 – 294,150	48,330	40,446 – 56,215	203,523	168,292 – 238,755
VRE	67,478	54,382 – 80,573	28,130	22,110 – 34,149	39,348	31,696 – 47,000
ESCR-EK	200,884	163,692 - 238,077	35,812	27,738 – 43,886	165,072	134,172 – 195,972
CRE	13,387	10,400 – 16,374	4,660	3,508 – 5,813	8,726	6,611 – 10,842
CRAsp	11,111	8,284 – 13,939	5,067	3,573 – 6,561	6,045	4,424 – 7,665
MDR *P. aeruginosa*	32,399	25,777 – 39,021	12,470	9,205 – 15,734	19,929	15,766 – 24,093

[a]The total number of cases does not equal the sum of the six individual pathogens due to deduplication of cases with both CRE and ESCR-EK resistance phenotypes.

© 2025 Wolford H et al. *JAMA Network Open*.

eFigure 2. National estimates of the sum of methicillin-resistant *Staphylococcus aureus*, vancomycin-resistant *Enterococcus* spp., extended-spectrum cephalosporin-resistant *Escherichia coli* and *Klebsiella* spp (excluding *Klebsiella aerogenes*) (ESCR-EK), carbapenem-resistant Enterobacterales, carbapenem-resistant *Acinetobacter* spp., and multidrug-resistant *Pseudomonas aeruginosa* stratified by location-onset and year with 95% confidence intervals, 2012-2022 A. cases per 10,000 hospitalizations B. case counts.

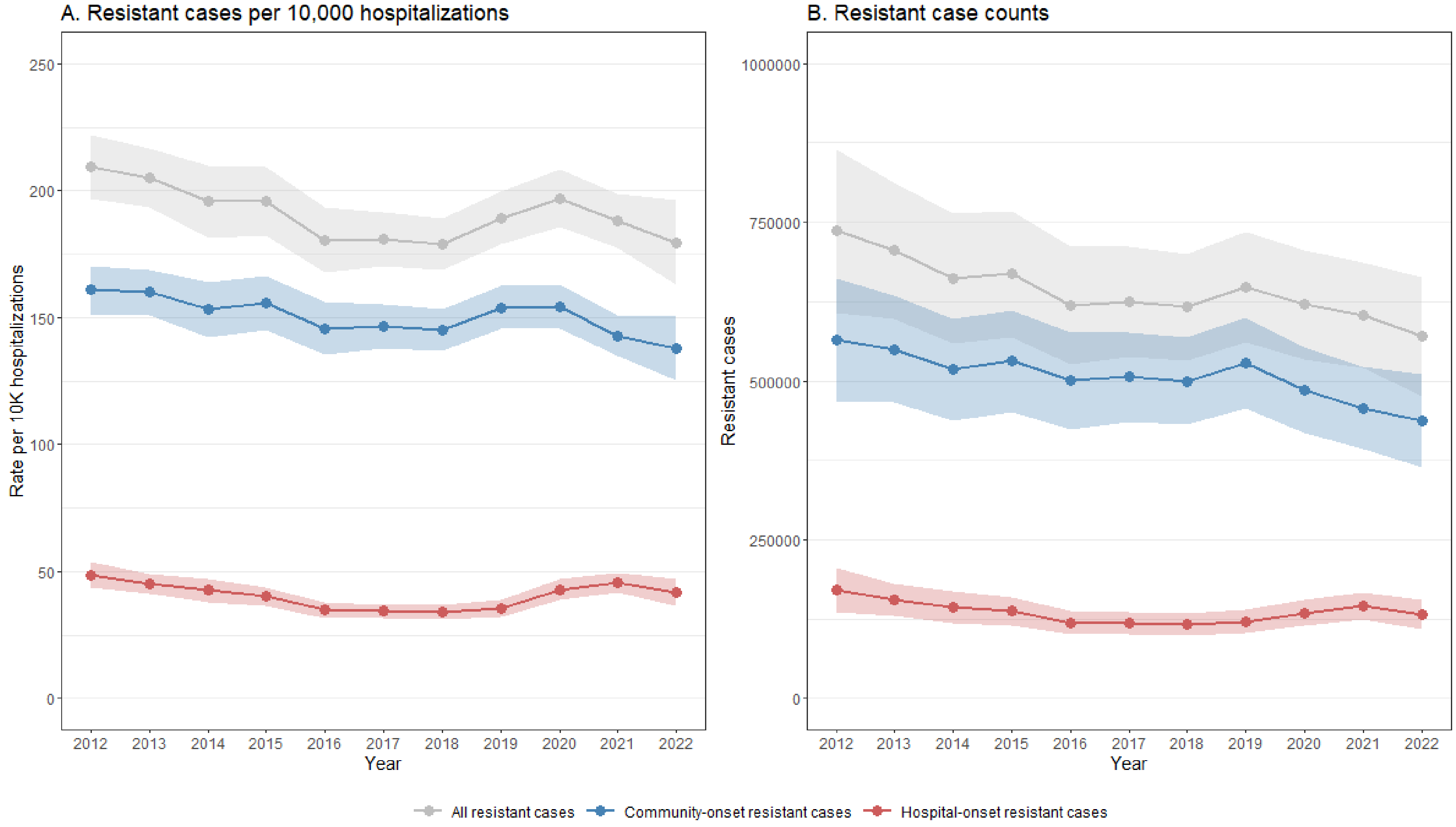

© 2025 Wolford H et al. *JAMA Network Open*.

eTable 4. National sum of methicillin-resistant *Staphylococcus aureus*, vancomycin-resistant *Enterococcus* spp., extended-spectrum cephalosporin-resistant *Escherichia coli* and *Klebsiella* spp. (excluding *Klebsiella aerogenes*) (ESCR-EK) suggestive of extended-spectrum β-lactamase production, carbapenem-resistant Enterobacterales (CRE), carbapenem-resistant *Acinetobacter* spp., and multidrug-resistant *Pseudomonas aeruginosa* cases per 10,000 hospitalizations; with 95% confidence intervals (CI), overall and stratified by location onset.

	2012	2013	2014	2015	2016	2017	2018	2019	2020	2021	2022
All resistant cases[a]	209.6	205.0	195.6	195.9	180.5	180.8	179.0	189.2	197.0	188.2	179.6
95% CI	197.0 - 222.1	193.2 - 216.7	181.3 - 209.8	182.4 - 209.4	167.7 - 193.4	170.1 - 191.5	168.9 - 189.2	178.8 - 199.7	185.4 - 208.5	177.5 - 198.9	163.1 - 196.1
Hospital-onset resistant cases	48.6	45.1	42.5	40.1	34.7	34.2	34.0	35.3	42.7	45.4	41.6
95% CI	43.7 - 53.6	41.2 - 48.9	37.9 – 47.0	36.5 - 43.8	31.7 - 37.8	31.5 – 37.0	31.0 - 36.9	31.9 - 38.6	38.6 - 46.8	41.5 - 49.3	36.2 - 47.1
Community-onset resistant cases	160.9	159.9	153.1	155.8	145.8	146.6	145.1	154.0	154.3	142.8	137.9
95% CI	151.5 - 170.4	150.8 - 168.9	142.2 - 163.9	145.0 - 166.6	135.2 - 156.3	137.7 - 155.4	136.9 - 153.3	145.7 - 162.2	145.4 - 163.2	134.7 - 150.9	125.3 - 150.5

[a]We deduplicated cases prior to estimating the total national burden as the same isolate could potentially count as a case for CRE and ESCR-EK phenotypes. Total resistant case rates will not align with the sum of the six individual case rates.

© 2025 Wolford H et al. *JAMA Network Open*.

eFigure 3. National estimated rates of hospital-onset (HO) antimicrobial-resistant cases per 10,000 hospitalizations, stratified by specimen body site (blood, urine, respiratory) and year with 95% confidence intervals, 2012-2022

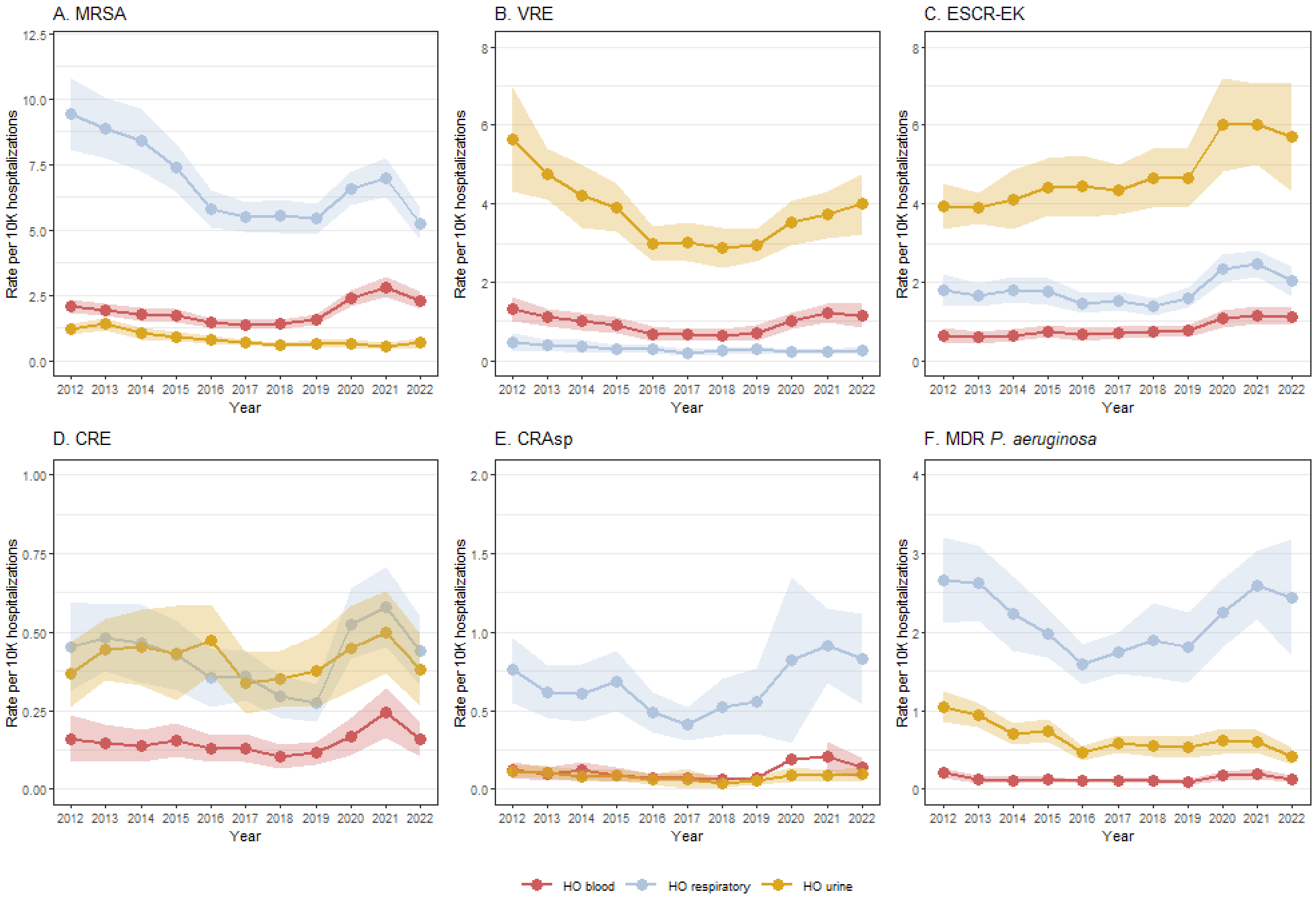

© 2025 Wolford H et al. *JAMA Network Open*.

eFigure 4. The percentage of hospital-onset (HO) resistant cases out of all HO cases tested for resistance, stratified by specimen body site (sterile/non-sterile) and year

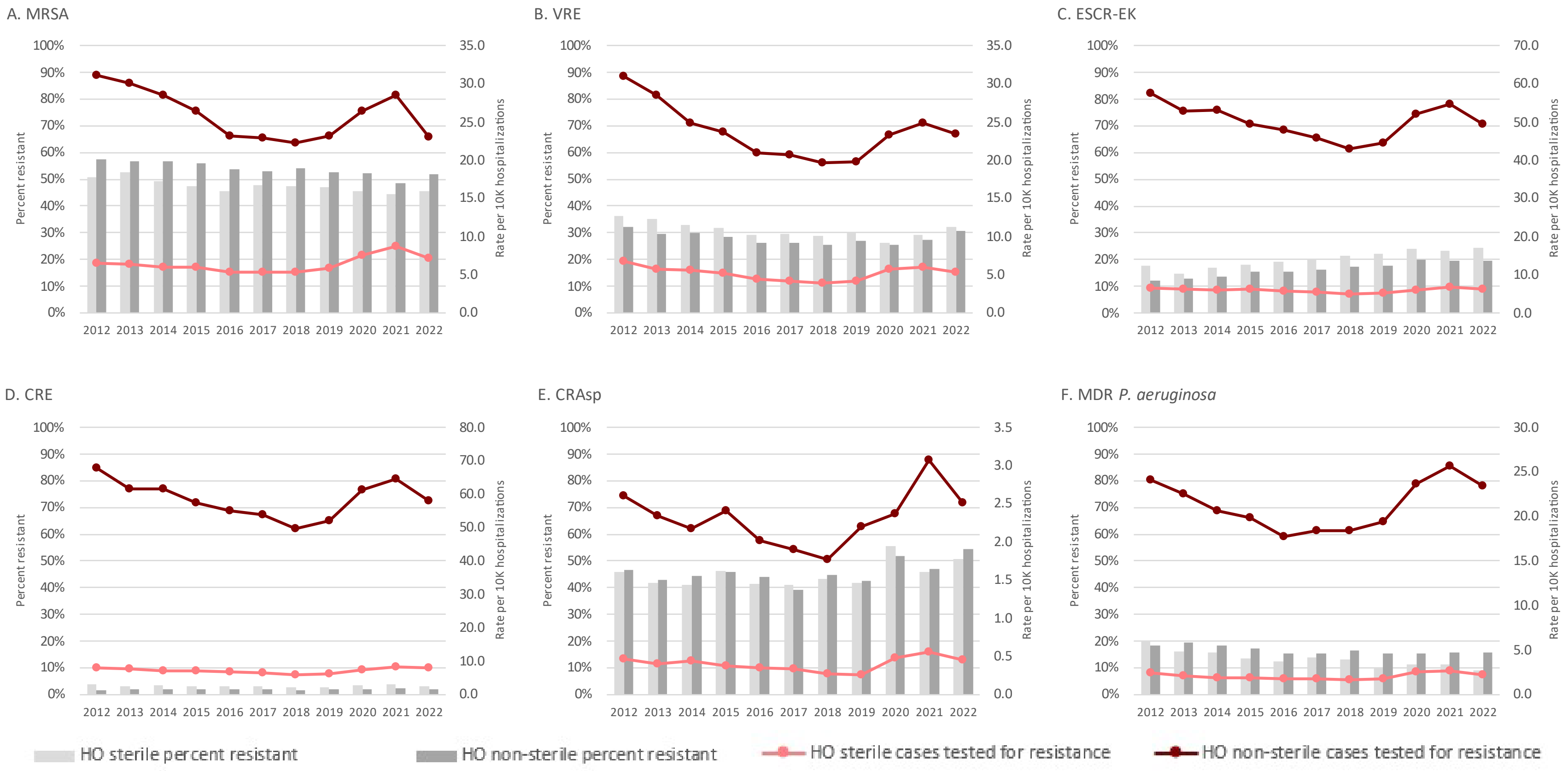

© 2025 Wolford H et al. *JAMA Network Open*.

eFigure 5. National estimated rates of antimicrobial-resistant cases per 10,000 hospitalizations for carbapenem-resistant Enterobacterales (CRE), extended-spectrum cephalosporin-resistant *Escherichia coli* and *Klebsiella* spp. (excluding *Klebsiella aerogenes*) (ESCR-EK) suggestive of extended-spectrum β-lactamase production, and vancomycin-resistant Enterococcus (VRE), stratified by species, location onset and year with 95% confidence intervals, 2012-2022

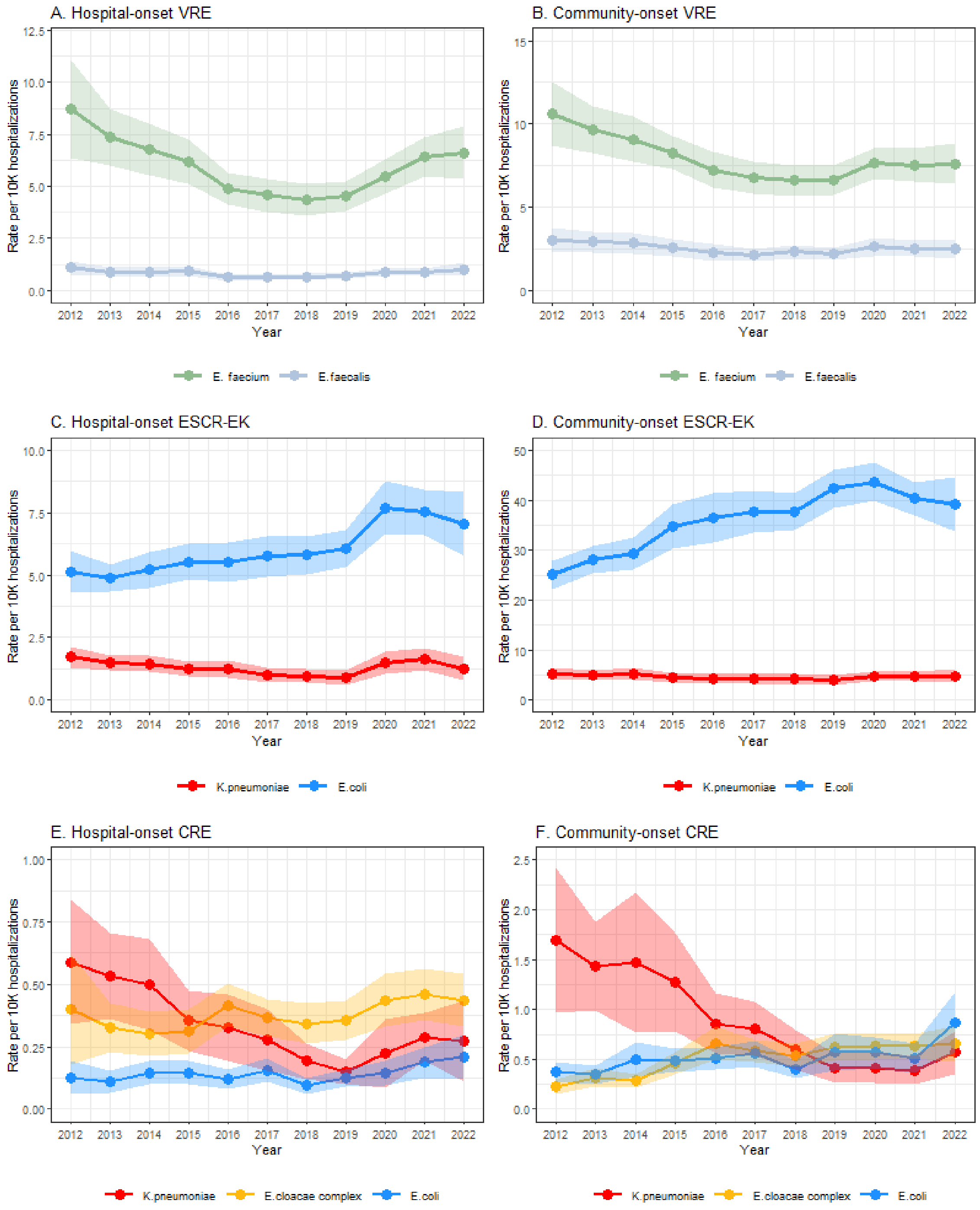

© 2025 Wolford H et al. *JAMA Network Open*.

eFigure 6. National estimated rates of hospital-onset (HO) antimicrobial-resistant cases per 10,000 hospitalizations by US census region, 2012-2022.

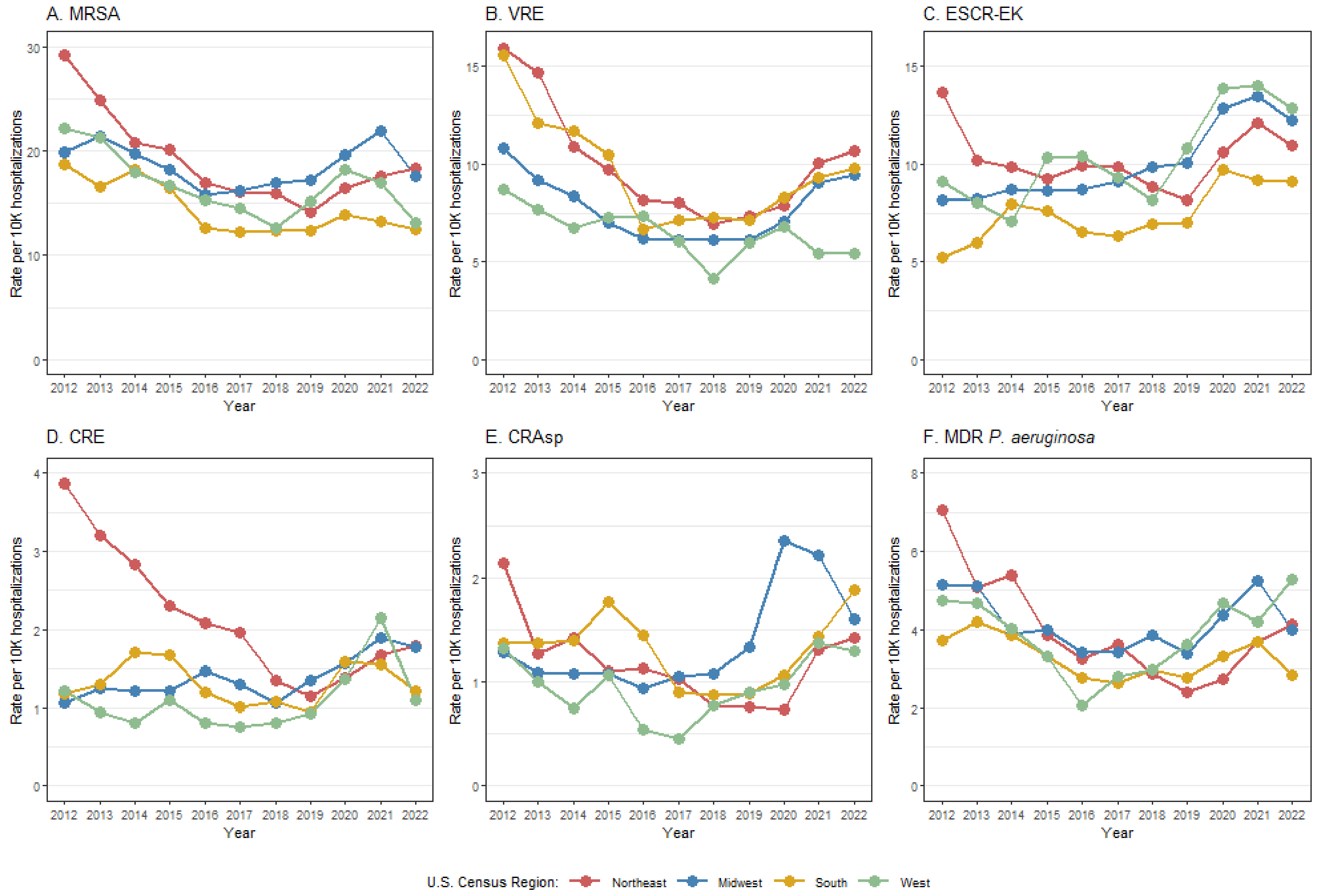

© 2025 Wolford H et al. *JAMA Network Open*.

eFigure 7. National estimated rates of community-onset (CO) antimicrobial-resistant cases per 10,000 hospitalizations, stratified by specimen body site (blood, urine, respiratory) and year with 95% confidence intervals, 2012-2022

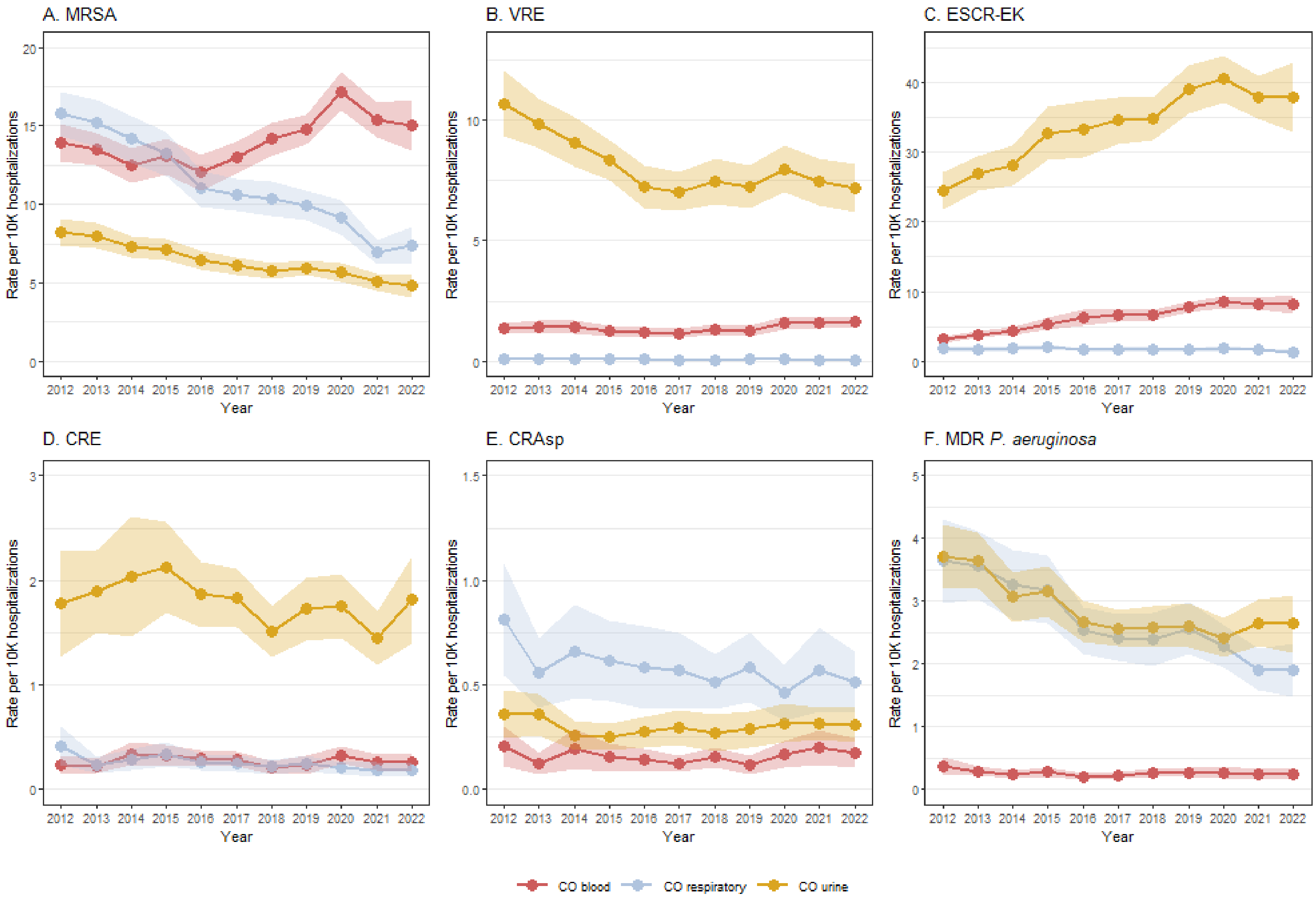

© 2025 Wolford H et al. *JAMA Network Open*.

eFigure 8. The percentage of community-onset (CO) resistant cases out of all CO cases tested for resistance, stratified by specimen body site (sterile/non-sterile) and year. Cases tested for resistance per 10,000 hospitalizations included in the figure.

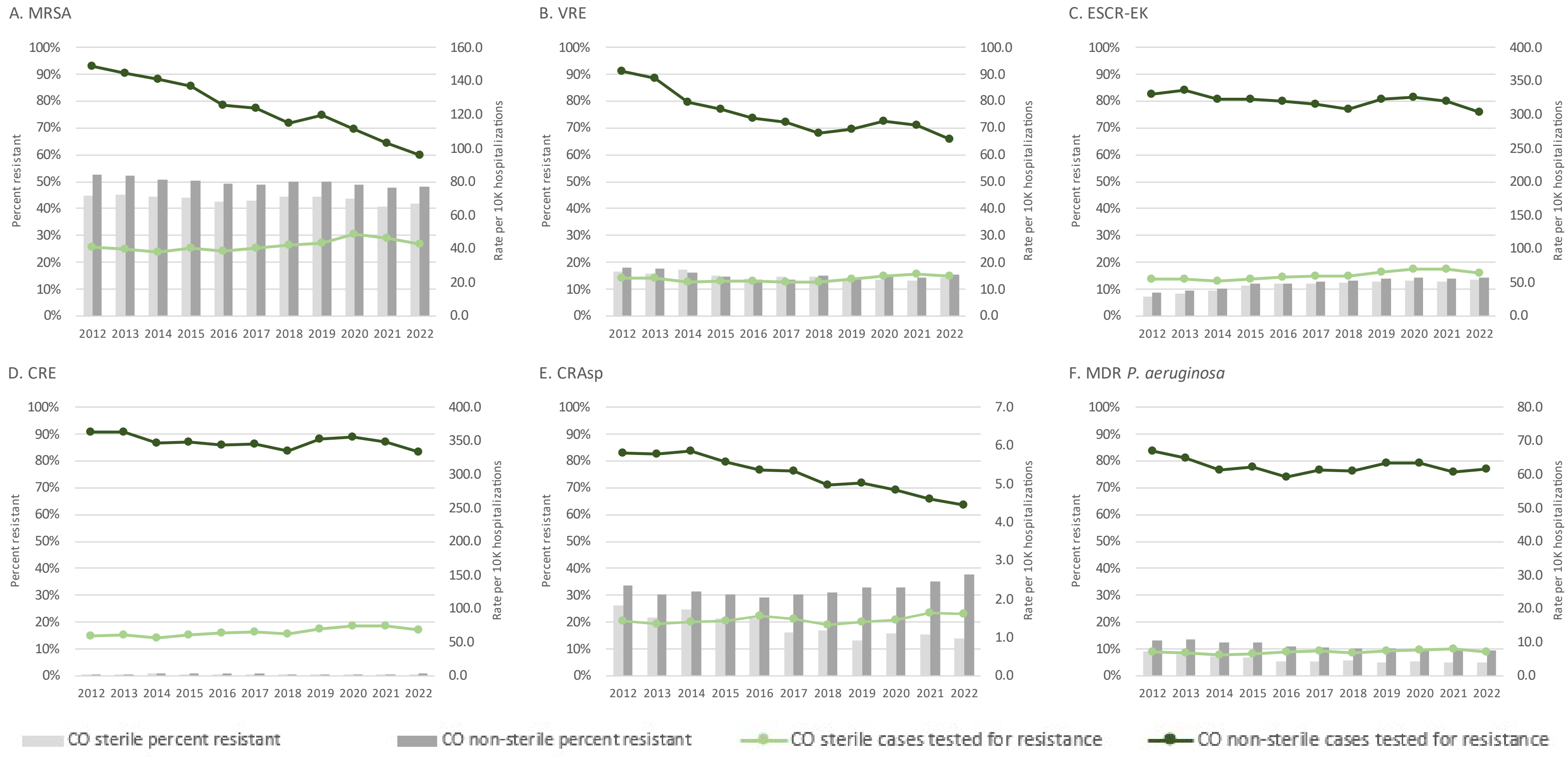

© 2025 Wolford H et al. *JAMA Network Open*.

eFigure 9. National estimated rates of community-onset (CO) antimicrobial-resistant cases per 10,000 hospitalizations, by US census region, 2012-2022.

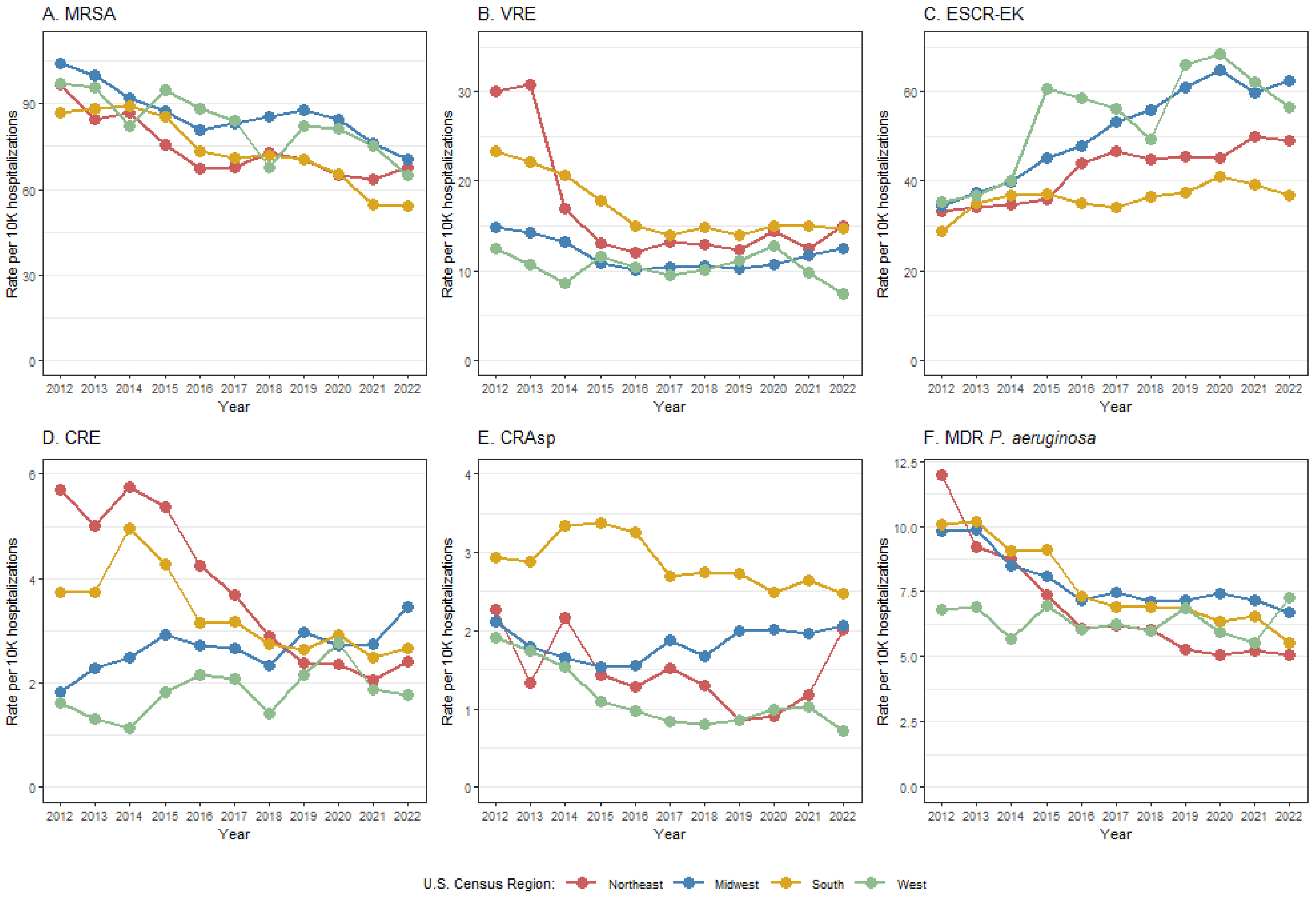

© 2025 Wolford H et al. *JAMA Network Open*.

eFigure 10. National estimated hospital-onset (HO) antimicrobial-resistant cases per 10,000 hospitalizations with 95% confidence intervals: Comparison between the dynamic hospital cohort and a cohort of consistent hospital reporters (379 hospitals reporting 48 months from 2018-2022)

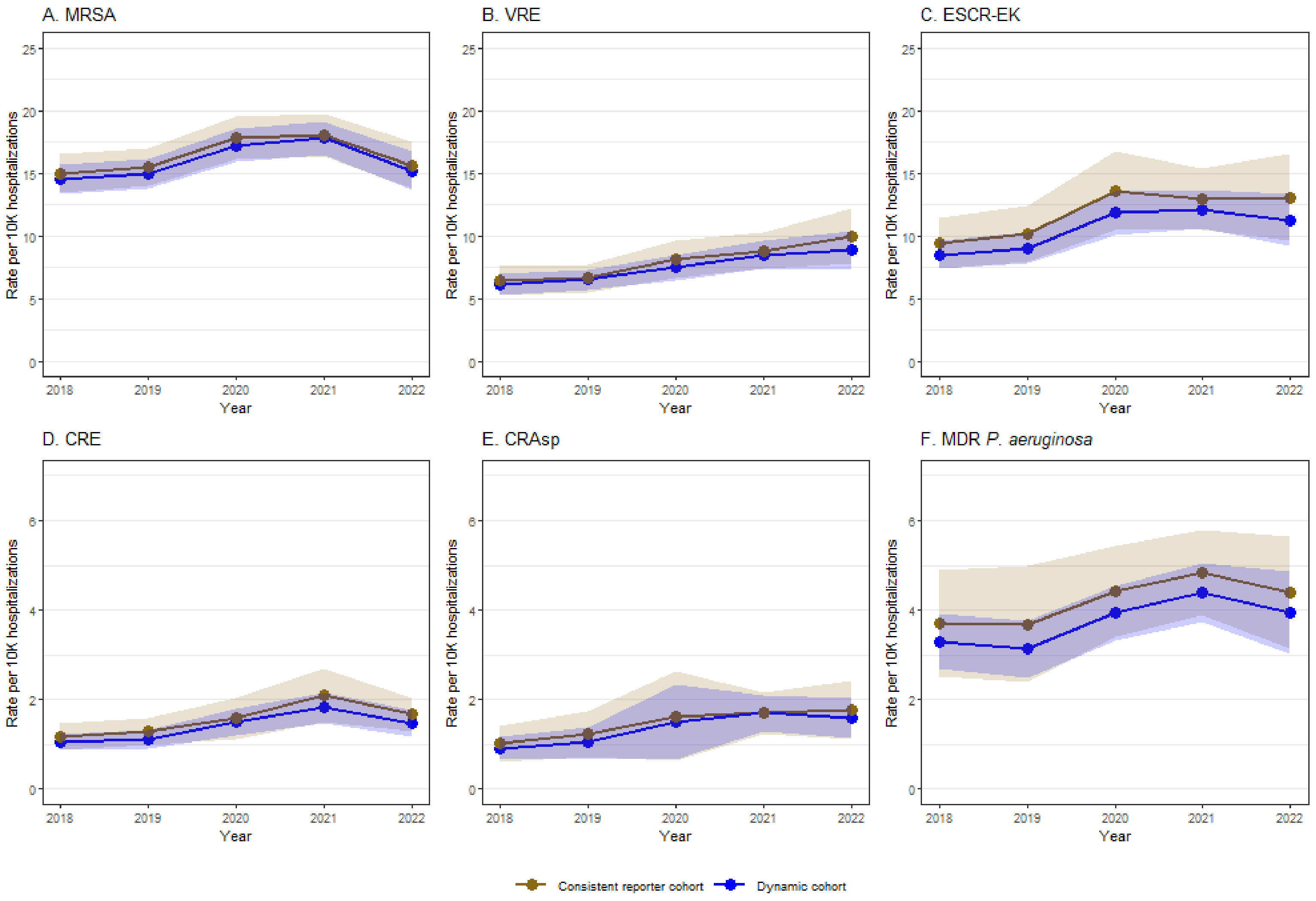

© 2025 Wolford H et al. *JAMA Network Open*.

eFigure 11. National estimated community-onset (CO) antimicrobial-resistant cases per 10,000 hospitalizations with 95% confidence intervals: Comparison between the dynamic hospital cohort and a cohort of consistent hospital reporters (379 hospitals reporting 48 months from 2018-2022).

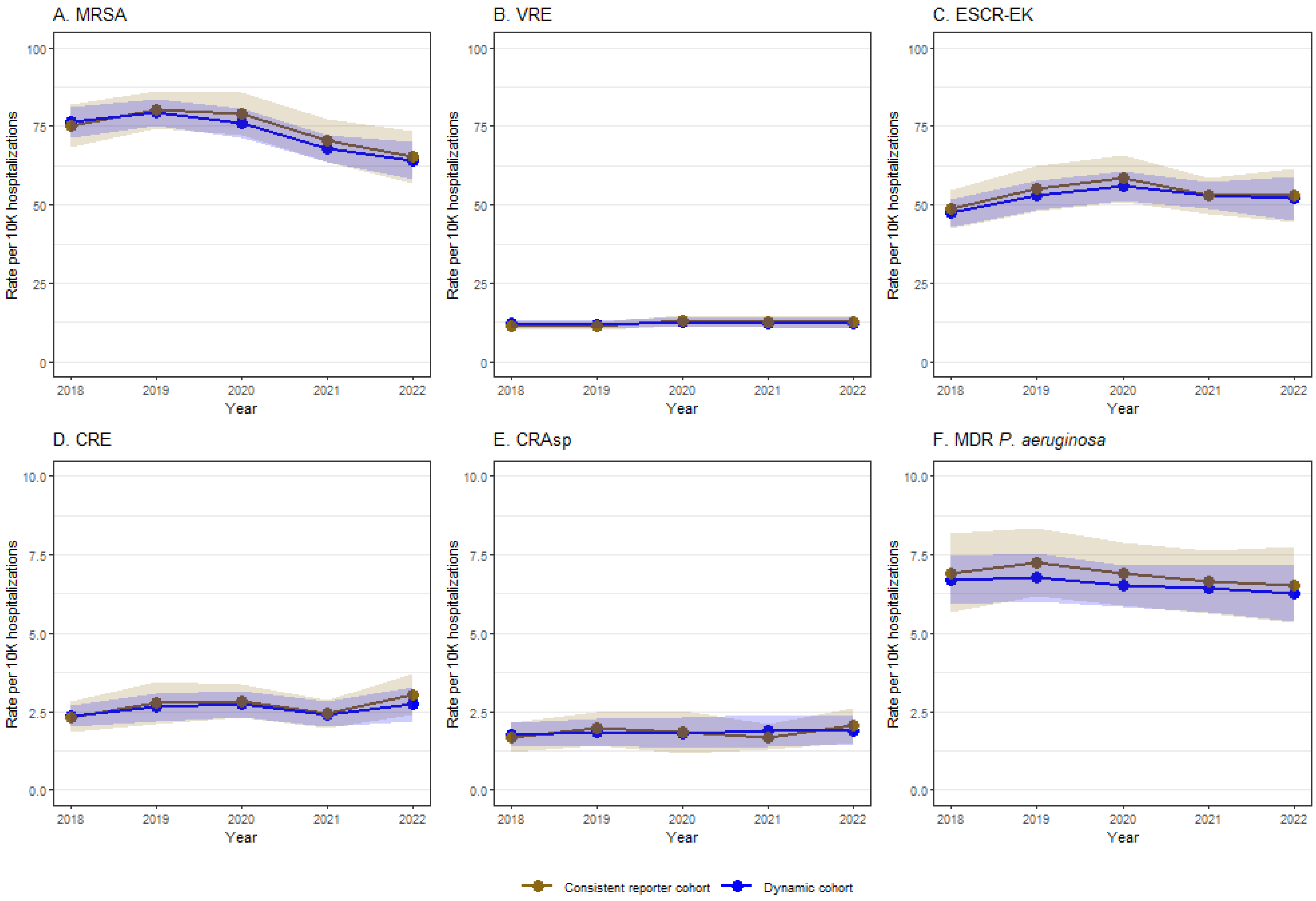

© 2025 Wolford H et al. *JAMA Network Open*.
