# Supplementary material for: Antimicrobial-Resistant Infections in Hospitalized Patients
Source: JAMA Netw Open. 2025 Mar 14;8(3):e2462059. doi: 10.1001/jamanetworkopen.2024.62059 (PMC11909612; doi:10.1001/jamanetworkopen.2024.62059)
Supplement: Supplement 3. — Data Sharing Statement [file jamanetwopen-e2462059-s003.pdf]

Data Sharing Statement

Wolford. Antimicrobial-Resistant Infections in Hospitalized Patients. *JAMA Netw Open*. Published February 27, 2025. doi:10.1001/jamanetworkopen.2024.62059

Data
Data available: No

Additional Information
Explanation for why data not available: Our analysis is based off proprietary datasets from PINC-AI Healthcare Database and the Becton Dickinson Insights Research Database.
